# Reprogramming Cells for Synergistic Combination Therapy with Nanotherapeutics against Uveal Melanoma

**DOI:** 10.3390/biomimetics3040028

**Published:** 2018-09-25

**Authors:** Paula Milán Rois, Alfonso Latorre, Ciro Rodriguez Diaz, Álvaro del Moral, Álvaro Somoza

**Affiliations:** Instituto Madrileño de Estudios Avanzados en Nanociencia (IMDEA Nanociencia), Centro Nacional de Biotecnología (CNB-CSIC) Associated Unit, Faraday 9, 28049 Madrid, Spain; paula.milan@imdea.org (P.M.R.); alfonso.latorre@imdea.org (A.L.); ciro.rodriguez@imdea.org (C.R.D.); delmoilex@gmail.com (Á.d.M.)

**Keywords:** uveal melanoma, microRNAs, SN38, gold nanoparticles, combination therapy

## Abstract

Uveal melanoma (UM) is the most common primary intraocular malignant tumor in adults and around half of the patients develop metastasis and die shortly after because of the lack of effective therapies for metastatic UM. Consequently, new therapeutic approaches to this disease are welcome. In this regard, microRNAs have been shown to have a key role in neoplasia progression and have the potential to be used as therapeutic tools. In addition, in different cancers including UM, a particular microRNA signature appears that is different from healthy cells. Thus, restoring the regular levels of microRNAs could restore the normal behavior of cells. In this study, four microRNAs downregulated in UM have been chosen to reprogram cancer cells, to promote cell death or increase their sensitivity to the chemotherapeutic SN38. Furthermore, to improve the internalization, stability and/or solubility of the therapeutic molecules employed in this approach, gold nanoparticles (AuNPs) were used as carriers. Remarkably, this study found a synergistic effect when the four oligonucleotides were employed and when the chemotherapeutic drug was added.

## 1. Introduction

Uveal melanoma (UM) is a malignant intraocular tumor with a high metastatic risk [1]. Unfortunately, the treatments for the metastatic UM are not effective [2] due to the unique characteristics of this tumor and because the visible clinical features only emerge at later stages of the disease [3,4]. Drugs developed for other tumors, such as Irinotecan [5,6], which is used for colon and lung tumors, have been used for UM. Furthermore, several chemotherapeutics agents are in clinical trials to treat UM, such as Selumetinib or AZD 8055. While these agents are selective against the mitogen-activated protein kinase (MAPK) and mammalian target of rapamycin (mTOR) pathways, respectively; they are not very selective and as a result, healthy cells are also affected.

Therefore, novel approaches to tackle this disease are desirable. In this sense, non-coding genetic elements, such as microRNAs, endogenous small interference RNAs (endo-siRNAs), long non-coding RNAs (lncRNAs), PIWI-associated small RNAs (piRNAs) and small nucleolar RNAs (snoRNAs), are being explored as new therapeutic targets since they play a major role in cellular events such as cell proliferation, differentiation, apoptosis or invasiveness capacity [7,8,9,10,11,12,13]. Interestingly, treatments based on these molecules are expected to achieve excellent selectivity and reduce side effects as compared to traditional treatments, because of the complementary sequence interactions involved in those approaches.

Among the most studied nucleic acids are microRNAs, which besides their therapeutic potential, can also be used to detect diseases as each tumor presents a unique microRNA profile depending on the tissue and cancer stage [14]. Additionally, several studies use their particular signature to classify the different diseases and use this information to apply the most suitable treatment [8,15].

MicroRNAs are transcribed initially as primary precursors (pri-microRNAs) from intron coding genes or intergenic regions, which are cleaved by the Drosha complex to produce the precursor hairpin molecule (pre-microRNA). These structures are transported by exportin-5 from the nucleus to the cytoplasm, where the Dicer complex processes pre-microRNAs into the mature microRNAs. Finally, the mature microRNAs guide the RNA-induced silencing complex (RISC) to their corresponding target messenger RNAs (mRNAs), promoting transcript reduction and/or inhibition [16].

MicroRNAs are dysregulated in many diseases such as Alzheimer’s, cardiac damage, and cancer [17,18,19,20,21,22]. Particularly in cancers such as sarcoma, glioblastoma, pancreatic cancer, breast and colon cancer, oncogenic microRNAs (e.g., microRNA-20a, microRNA-9, microRNA-21) are overexpressed [23] and/or tumor suppressors microRNAs (e.g., microRNA-145, microRNA-204) are downregulated [24].

In the present study, microRNA-34a, microRNA-182, microRNA-137 and microRNA-144 have been selected since they are downregulated in UM and other cancers [25]. In particular, they are used in combination as this could provide better results, when compared to the use of individual microRNAs [26]. Regarding their roles, microRNA-34a is a proapoptotic transcriptional target of the p53 tumor-suppressor gene effector network. Its primary target is the c-Met mRNA [27], which is a tyrosine-kinase receptor implicated in the pathways of son of sevenless homolog (SOS), phosphatidylinositol 3-kinase (PI3K), and the Ras protein family [28]. Moreover, overexpression of microRNA-34a can downregulate the Akt protein and cell cycle-related proteins, reducing the metastatic and proliferation potential of the cells [27].

MicroRNA-182 participates in the tumor suppression network of p53 in UM, where melanogenesis associated transcription factor (MITF), B-cell lymphoma 2 (BCL2) and cyclin D2 are its main targets. MITF is a master regulator of melanoma oncogenes that mediates the inhibition of Akt and extracellular signal-regulated kinases ERK1/2 pathways, in addition to controlling c-Met. Furthermore, microRNA-182 controls the forkhead box protein O1 (FoxO1), which is related to osteogenesis, T-cell development and tumorigenesis [29].

MicroRNA-137 has three major targets: MITF, and the cyclin-dependent kinases CDK2 and CDK6. It is a potent suppressor of the p160 steroid receptor co-activator (SRC) family of transcriptional co-activators, which can inhibit SRC-mediated, steroid receptor-dependent and -independent transcription [30]. Other studies identified C-terminal binding protein 1 (CTBP1) as a microRNA-137 target. By regulating CTBP1 through microRNA-137, it is possible to avoid E-cadherin suppression [31].

MicroRNA-144 acts as a tumor suppressor in UM, inhibiting cell proliferation and migration. As with the previously mentioned microRNAs, c-Met is one of its potential targets [32]. 

To restore the normal levels of microRNAs in tumoral cells, vectors encoding those microRNAs or whole microRNAs can be added to cells. However, modified nucleic acids that mimic the role of the microRNAs (microRNAs mimics) are usually employed at the preclinical and clinical level [26,33], such as small interference RNAs (siRNAs), which must be modified to increase their stability in serum and achieve the required activity [34]. A related strategy employs the use of antisense technologies, which are made of deoxyribonucleotides. Such DNA-based microRNA mimics are more stable and cheaper than their RNA counterparts, and despite their lower activity, they can be used in combination with chemotherapeutic agents with remarkable results [35,36]. Despite the enormous potential of oligonucleotides as therapeutics, they present some disadvantages, mainly when based on RNA, as outlined below.

1. Low stability. They can be degraded by blood circulating enzymes [37], reducing their therapeutic activity [17].
2. Poor cell internalization. Their negative charge prevents passive endocytosis [17].
3. Poor cellular selectivity. The oligonucleotides cannot discriminate between healthy and cancer cells, leading to a non-specific delivery [37].

To overcome these problems, delivery systems such as micelles, liposomes, dendrimers, inorganic particles, nanotubes and carbon nanoparticles, nanoemulsions, viral nanocarriers, polymeric or peptide nanoparticles and solid lipid nanoparticles are being evaluated [38,39,40,41,42,43,44,45,46]. 

Among the different systems, gold nanoparticles (AuNPs) are excellent nanomaterials for this purpose, since they are easy to prepare and modify and are considered non-toxic when properly modified. In particular, when functionalized with oligonucleotides these structures do not affect the gene expression or viability of cells. In this case, when the AuNPs are densely modified with oligonucleotides, they are known as spherical nucleic acids (SNAs), which, among other properties, can translocate easily in a wide variety of cell lines [47,48,49].

Despite the great therapeutic potential of oligonucleotides, chemotherapy-based treatments are usually employed to treat cancers. Unfortunately, the associated lack of selectivity, drug resistance, and high toxicity make this approach far from ideal and limits its use [50]. Indeed, monotherapy has been proven ineffective for cancer treatment due to the development of resistance and metastasis. For this reason, combination therapies are the preferred choice, since it is possible to reduce the amount of the most toxic agent [51] and reduce relapse. In this sense, the combination of microRNAs and chemotherapeutic drugs has provided excellent results in different type of cancers, such as human glioma [26].

In this study, we have used SN38 (7-ethyl-10-hidroxycamptothecin), a topoisomerase I inhibitor [52] in combination with oligonucleotides, which are designed to mimic the role of the above-mentioned microRNAs. SN38 has not been tested in UM, although there are reports on Irinotecan, its prodrug, which presents 200–2000-fold less activity [53]. The principal drawback of SN38 is its poor aqueous solubility (<5 μg/mL), which prevents systemic administration [54]. To overcome this limitation, drug delivery systems based on liposomes have been reported [53]. In this regard, we envision that the conjugation of therapeutic oligonucleotides and SN38 on AuNPs can overcome the inherent limitations of these molecules and provide a more effective therapy. 

## 2. Materials and Methods

### 2.1. Materials

Solvents and chemical reagents were purchased from Sigma-Aldrich (San Luis, MO, USA), abcr GmbH (Karlsruhe, Germany), Thermo Fisher Scientific (Waltham, MA, USA), Scharlab (Sentmenat, Barcelona, Spain), FluoroChem (Hadfield, UK) and VWR (Radnor, PA, USA).

Oligonucleotides were purchased from Integrated DNA Technologies (IDT, Coralville, IA, USA).

Roswell Park Memorial Institute (RPMI) medium, streptomycin–penicillin (100X), fetal bovine serum (FBS), l-glutamine (100X), trypsin (10X), phosphate-buffered saline (PBS) and cell culture plasticware were purchased from VWR. Thiazolyl blue tetrazolium bromide, propidium iodide (PI), Hoechst 33342 and dimethyl sulfoxide (DMSO) were obtained from Sigma-Aldrich; and Lipofectamine 2000, RNase H and Opti-MEM from Thermo Fisher Scientific. c-Met and anti-rabbit IgG Alexa Fluor 488 antibodies were purchased from Cell Signaling Technology (Danvers, MA, USA).

### 2.2. Modified Oligonucleotides

The sequences of the oligonucleotides employed are described in Table 1. siRNA duplexes were obtained after the annealing of complementary sequences using an annealing buffer described in Section 2.3.1. The aptamer AS1411 was prepared as previously reported in a Mermade 4 DNA synthesizer [47]. 

### 2.3. Oligonucleotide Solution Preparation

#### 2.3.1. RNA Annealing (siRNAs)

siRNA duplexes were obtained by mixing the guide and the passenger oligonucleotides in an annealing buffer. The annealing buffer was prepared following the Sigma-Aldrich protocol for annealing oligonucleotides [55].

siRNA-34a was obtained from oligonucleotides 9 and 10; siRNA-182 was obtained from 11 and 12; siRNA-137 was obtained from 13 and 14 and siRNA-144 was obtained from 15 and 16 (Table 1).

#### 2.3.2. siRNA Mix

A sample containing the four siRNAs previously prepared was obtained by mixing equimolar quantities of each siRNA. 

#### 2.3.3. DNA Mix

DNA mix-1 (34.04 μM, final concentration) was obtained from equimolar amounts of oligonucleotides 1, 2, 3 and 4 (Table 1) in water. This mixture contains unmodified oligonucleotides. 

DNA mix-2 (20 μM, final concentration) was obtained from equimolar amounts of oligonucleotides 5, 6, 7 and 8 (Table 1) in water. This mixture contained thiol-modified oligonucleotides and was used for the functionalization of AuNPs.

### 2.4. Gold Nanoparticles Synthesis and Characterization

The gold nanoparticles were prepared following standard reported procedures [56]. The AuNPs concentration was calculated using the Beer–Lambert law [57] from the absorbance recorded at 520 nm and the extinction coefficient of 2.7 × 10^8^ for 13 nm nanoparticles [58]. 

Particle size and shape were examined by transmission electron microscopy (TEM) in a JEOL JEM 1010 (JOEL, Akhisima, Japan), operating at 100 kV. Samples were prepared by placing one drop of a dilute suspension onto a carbon-coated copper grid and drying the drop with paper after 2 min. The size distributions were determined through manual analysis of ensembles of over 200 particles found in randomly selected areas of the enlarged micrographs, with Image J software [59] to obtain the mean size and standard deviation (Supplementary Figure S1).

#### Gold Nanoparticle Functionalization

The bare nanoparticles were modified with the different components using the conditions summarized in Table 2. The oligonucleotides (DNA mix 2 and siRNA mix) were previously treated for 1 h with tris(2-carboxyethyl) phosphine hydrochloride (TCEP), to remove the protecting groups on the thiol moieties. After the addition of each reagent, the solution was incubated for 30 min. The procedure was done as follows: The reagents were added to 1 mL of a solution of AuNPs (10 nM) in the order and quantities detailed in Table 2. The last step involves the addition of NaCl (70 μL, 5 M) and incubation with agitation at room temperature overnight. The particles were centrifuged down (300× *g*) at 4 °C, the supernatant removed and resuspended in water. The process was repeated three times to remove the unbound material [60].

The amount of oligonucleotide on the nanoparticle was determined as follows: after purification of the nanoparticle the supernatants were dried in an Eppendorf Concentrator plus (V-AQ mode) (Eppendorf, Hamburg, Germany) for 5 h; then the sample was diluted in water (1 mL) and the concentration of oligonucleotides quantified by absorbance at 260 nm in a plate reader Synergy H4 Hybrid reader (BioTEK, Winooski, VT, USA). This amount was subtracted from the amount added. Concentrations of AuNP–DNA mix and AuNP siRNA mix were 3.9 and 3.28 μM, respectively. The quantification of SN38 on the nanoparticle was carried out in the same way as above, but the absorbance was recorded at 380 nm, resulting in a concentration of 10 μM.

The hydrodynamic size, by dynamic light scattering (DLS), and zeta-potential of the AuNPs was measured on a Zeta Sizer Nano-ZS (Malvern Instruments, Worcestershire, UK). The studies were performed in 12 runs, in a standard cuvette at 25 °C. The AuNPs were diluted in 1 mL water (pH 5.8) to a final concentration of 1 nM. The AuNP’s refraction index for a spherical particle was 1.330. The data presented is the average distribution of these measurements as a function of the number of particles expressed as a percentage.

### 2.5. Cell Culture and Viability Studies

Mel 202 cells were cultured (Mel202 were donated by Susana Ortiz-Urda at the University of California, San Francisco, CA, USA) in RPMI medium with 10% FBS, 1% streptomycin–penicillin and 1% l-glutamine at 37 °C in a Binder CB210 incubator (5% CO_2_). All the procedures were performed inside a laminar flow hood Telstar CV-30/70 (Telstar, Terrassa, Spain). Cells were grown in 24-well plates (30,000 cells/well).

The experiments were done when cells reached 60% confluency.

#### 2.5.1. alamarBlue Viability Assay

A stock solution of resazurin sodium salt (Sigma-Aldrich, St. Louis, MO, USA) (1 mg/mL) in PBS was diluted 1% (*v*/*v*) in complete RPMI medium and added to the cells. After 3 h in the incubator (37 °C), the fluorescence was measured at 25 °C in a plate reader Synergy H4 Hybrid reader (BioTEK), λ_ex_ = 550 nm, λ_em_ = 590 nm.

The fluorescent intensity measurements were processed using the following Equation:

% Cell viability = ((Sample data − Negative control)/(Positive control − Negative control)) × 100

The positive control corresponds with untreated cells. A resazurin solution without cells was used as negative control.

#### 2.5.2. Oligonucleotide Transfection

A total of 50 μL Opti-MEM was mixed with oligonucleotides (14, 15, 16, 17) or DNA mix 1. Next, it was mixed with 1 μL lipofectamine 2000 in 50 μL Opti-MEM. The final mixture was incubated for 20 min and then 100 μL of the final mixture was added to cells. The final concentration of 14, 15, 16, 17 or DNA mix 1 was 140 nM. After 24 h, the cells were washed three times with PBS and RPMI medium was added.

#### 2.5.3. Chemotherapy

SN38 stock solution was prepared at 100 μM in DMSO. Then different concentrations of SN38 (100, 50, and 25 nM) were prepared in RPMI medium. It was incubated for 24 h with the cells, then washed three times with PBS and RPMI medium was added. After an additional 24 h, the viability assay was carried out as described in Section 2.5.1. 

#### 2.5.4. Combination Treatment

In this case, the oligonucleotides were transfected, as indicated in Section 2.5.2. After 24 h the cells were washed three times with PBS. Then SN38 (25 nM/well) was added and incubated for an additional 24 h. Then, the cells were washed with PBS and RPMI medium was added. The cell viability was evaluated after their incubation for an additional 24 h.

#### 2.5.5. Nanoparticles Treatment

A volume of 100 μL functionalized AuNPs were added in Opti-MEM (500 μL, total volume). The cells were incubated for 24 h, washed with PBS and RPMI medium was added. Twenty-four hours later, the viability was assessed, as described in Section 2.5.1.

### 2.6. c-Met Immunofluorescence

Cells were treated with the oligonucleotides as described in Section 2.5.2 and an immunofluorescence staining was performed as reported elsewhere [59] using an anti-c-Met antibody (Ab 1003, Cellular Signaling Technology, Danvers, MA, USA) (1:200) as a primary antibody and a goat anti-rabbit IgG Alexa 488 (1:200) as a secondary antibody. The nucleus was stained with Hoechst 33342 and fluorescence was examined using a Leica DMI3000 M inverted microscope (Leica, Wetzlar, Germany) at 274.29 exposure units. Images were collected and analyzed using a customized script for Fiji [61]. 

### 2.7. Flow Cytometry

Cells were harvested in 6-well plates at a 60% confluency and the treatment was carried out as described above. Then, the samples were trypsinized, fixed with paraformaldehyde 1% (*v*/*v*), washed with PBS and centrifuged at 177× *g* for 5 min in an Eppendorf centrifuge 5804 R (Eppendorf, Hamburg, Germany). Each sample was treated with 10 μg RNAsa A and 20 μg PI. Cell cycle analysis was performed in a Beckman Coulter Cytomics 500 Flow Cytometer (Beckman Coulter, Indianapolis, IN, USA) using 20,000 cells. The acquired data was analyzed with Multicycle software (Perttu Terho, Turku Centre for Biotechnology, Turku, Finland). 

These experiments were performed in the Flow Cytometry Service at the CNB-CSIC.

### 2.8. Synthesis of Modified SN38

**Scheme 1:**
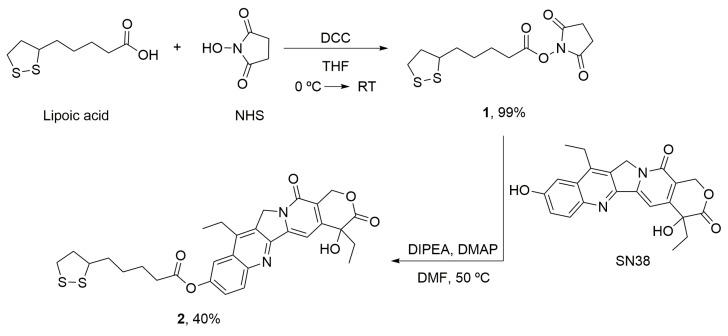
Synthesis of the modified SN38 (**2**) with the linker for the conjugation with AuNPs. Lipoic acid was activated (**1**) and incubated with SN38 to yield the desired product (**2**). DCC: *N*,*N*’-dicyclohexylcarbodimide; DIPEA: *N*,*N*-diisopropylethylamine; DMAP: 4-Dimethylaminopyridine; DMF: Dimethylformamide; NHS: *N*-hydroxysuccinimide; RT: Room temperature; THF: Tetrahydrofuran.

All reactions (Scheme 1) were monitored by thin-layer chromatography (TLC), which was performed on sheets of silica gel 60 F254 (Sigma-Aldrich). The products were purified by flash column chromatography using silica gel (60 Å, 230 × 400 mesh). Nuclear magnetic resonance (NMR) spectra were recorded on a Bruker Instrument (Bruker, Mannheim, Germany) and reported in MHz as solutions in CDCl_3_, the chemical shifts are reported in parts per million (ppm), and the coupling constants are reported in Hz. 

#### 2.8.1. α-Lipoic Acid–NHS (**1**)

Lipoic acid (1 g, 1 equiv) and *N*-hydroxysuccinimide (NHS) (667 mg, 1.2 equiv) were dissolved in tetrahydrofuran (THF) (20.8 mL), and the solution was stirred at 0 °C for 10 min. A solution of *N*,*N*’-dicyclohexylcarbodimide (DCC) (1.2 g, 1.2 equiv) in THF (1.7 mL) was added slowly to the lipoic acid and NHS solution. The reaction was stirred at room temperature for 5 h. The mixture was filtered, and the solution was kept in the freezer overnight, it was filtered again and the solvent was eliminated in vacuum. The compound **1** (Scheme 1) was obtained as yellow oil (1.43 g, 99% yield) [62]. ^1^H NMR (400 MHz, CDCl3): δ = 3.60–3.50 (m, 4H), 3.20–3.06 (m, 1H), 2.81 (s, 4H), 2.61 (t, *J* = 7.4 Hz, 2H), 2.50–2.36 (m, 1H), 1.94–1.88 (m, 1H), 1.82–1.72 (m, 2H), 1.72–1.66 (m, 2H), 1.62–1.46 (m, 2H) (Supplementary Figure S2). ^13^C NMR (101 MHz, CDCl3): δ = 169.13, 168.42, 67.42, 40.15, 38.52, 34.42, 33.21, 30.79, 25.59, 22.59, 24.36, 23.39.

#### 2.8.2. α-Lipoic Acid–SN38 (**2**)

Compound **1** (56 mg, 2 equiv), SN38 (36 mg, 1 equiv) and 4-dimethylaminopyridine (DMAP) (3 mg) were dissolved in dimethylformamide (DMF) (3.7 mL), then *N*,*N*-diisopropylethylamine (DIPEA) was added to the solution. The reaction was stirred at 50 °C for 18 h. The solvent was eliminated in vacuum and the crude was purified by flash chromatography (CH_2_Cl_2_ → CH_2_Cl_2_/MeOH 40:1) to obtain the compound **2** (Scheme 1) as white-yellow solid (21 mg, 40% yield). ^1^H-NMR (400 MHz, CDCl_3_): δ = 8.24 (d, *J* = 9.2 Hz, 1H), 7.83 (d, *J* = 2.5 Hz, 1H), 7.65 (s, 1H), 7.55 (dd, *J* = 9.2, 2.5 Hz, 1H), 5.76 (d, *J* = 16.3 Hz, 1H), 5.35–5.29 (m, 1H), 5.26 (s, 2H), 3.68–3.59 (m, 1H), 3.25–3.10 (m, 4H), 2.69 (t, *J* = 7.4 Hz, 2H), 2.55–2.46 (m, 1H), 2.00–1.93 (m, 1H), 1.90–1.83 (m, 3H), 1.83–1.76 (m, 2H), 1.70–1.59 (m, 3H), 1.40 (t, *J* = 7.7 Hz, 3H), 1.04 (t, *J* = 7.4 Hz, 3H) (Supplementary Figure S3). ^13^C-NMR (101 MHz, CDCl3): δ = 173.95, 171.85, 157.67, 151.94, 150.18, 149.64, 147.49, 146.95, 145.27, 132.13, 127.48, 127.27, 125.41, 118.55, 114.59, 97.98, 72.76, 66.38, 56.34, 49.40, 40.29, 38.55, 34.61, 34.21, 31.62, 28.75, 24.58, 23.19, 14.01, 7.83 (Supplementary Figure S4).

### 2.9. Statistical Analysis

The statistical analysis was performed in R Project for Statistical Computing (R-3.2.5) software [63]. One-way analysis of variance (ANOVA) was used to compare the mean value of each condition vs. control. Significant differences between the means were accepted when the *p*-value was lower than 0.001 (***).

## 3. Results

### 3.1. DNA-Based microRNA Mimics

**Figure 1:**
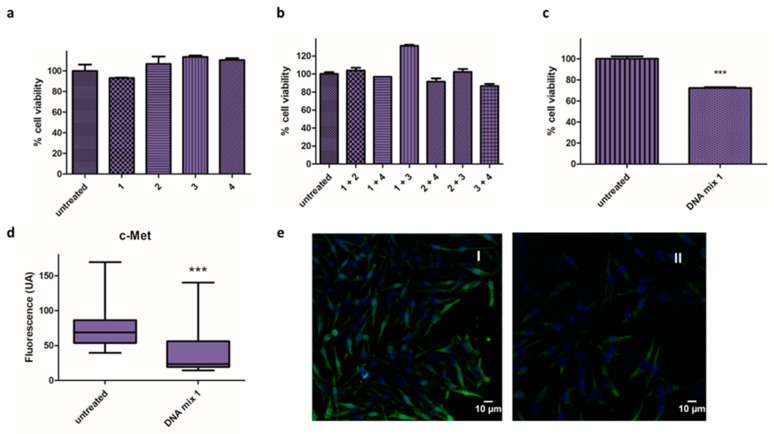
Activity of microRNA mimics. (**a**–**c**) Cell viability assay in Mel 202 cells using different combination of oligonucleotides 1, 2, 3 and 4: (**a**) Mel 202 cells were transfected with individuals oligonucleotides (1, 2, 3 and 4) treatment at 140 nM/well; (**b**) Mel 202 cells were transfected with oligonucleotides in pairs (final oligonucleotide concentration of 140 nM); (**c**) Mel 202 cells were transfected with DNA mix 1 at 140 nM (*** *p* < 0.001). (**d**,**e**) Immunofluorescence analysis in Mel 202 cells: (**d**) Fluorescence intensity (arbitrary units, AU) of c-Met in untreated cells and treated with the DNA mix 1 (*** *p* < 0.001); (**e**) Representative immunofluorescence images of cells untreated (I) and treated with the DNA mix 1 (II). c-Met is shown in green and nucleus are labeled in blue by Hoechst staining. Statistical analysis was performed using one-way ANOVA (each group vs. control).

DNA-based microRNA mimics 1, 2, 3, and 4 (Table 1) were transfected individually into Mel 202 cells and cell viability was assessed using the alamarBlue assay after 48 h. The reduction in cell viability was negligible in all cases (Figure 1a), and the effect of the microRNA mimics when combined was evaluated, both in pairs (Figure 1b) and all together in DNA mix 1 (Figure 1c). Interestingly, a 30% reduction in cell viability was observed in the latter conditions.

In addition, the effect of the mixture on the expression of c-Met was analyzed by immunofluorescence (Figure 1d,e). Remarkably, the images revealed significant changes in fluorescence when the cells were treated with the DNA mix, precisely a 67% reduction in the expression of c-Met.

### 3.2. Combination Therapy

Since the DNA mix 1 provided a cytotoxic effect and reduced c-Met expression, we decided to study the effect in combination with the chemotherapeutic drug SN38. The siRNA mix was also evaluated in combination with SN38, since a higher reduction in cell viability could be expected.

**Figure 2:**
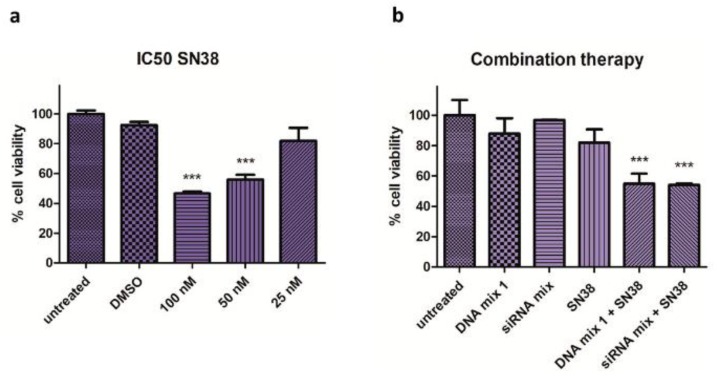
Half maximal inhibitory concentration (IC50) of SN38 and combination therapy effect. (**a**) Cell viability assay in Mel 202 cells treated with SN38 at different concentrations. The IC50 in which the cell viability is reduced 50% is 100 nM for Mel 202 cells (*** *p* < 0.001). (**b**) Cell viability assay in Mel 202 cells treated with SN38, DNA mix 1 and siRNA mix. The cell viability is reduced by 50% at a very low concentration of SN38 in the combination conditions (*** *p* < 0.001). Statistical analysis was performed using one-way ANOVA (each group vs. control).

First, the half maximal inhibitory concentration (IC50) of SN38 (100 nM) was assessed in Mel 202 cells (Figure 2a), and then the combined effect of the SN38 with DNA mix 1 and siRNA mix were examined (Figure 2b). A significant reduction in cell viability (50%) was observed using low concentrations of the drug (25 nM) and the DNA mix 1 or siRNA mix.

**Figure 3:**
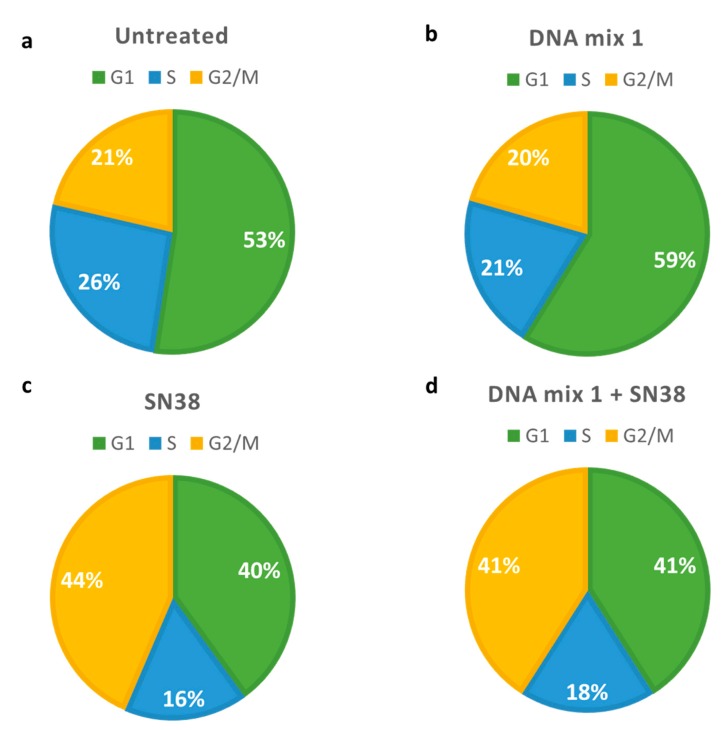
Pie charts describing flow cytometry data in Mel 202 cells. (**a**) Untreated cells; (**b**) Cells treated with 140 nM DNA mix 1; (**c**) Cells treated with 25 nM SN38; (**d**) Cells treated with 140 nM microRNA mimics and 25 nM SN38. Green: cells in G1 phase; blue: cells in S phase; yellow: cells in G2/M phase.

With the aim to study the potential effect of this combination on the cell cycle (Figure 3), cells were harvested after the treatment and analyzed by flow cytometry. The effect of DNA mix 1 was negligible (Figure 3b) as compared with the effect obtained with SN38, which significantly increases G2/M phase cells (Figure 3c).

### 3.3. Gold Nanoparticles as Carriers

**Figure 4:**
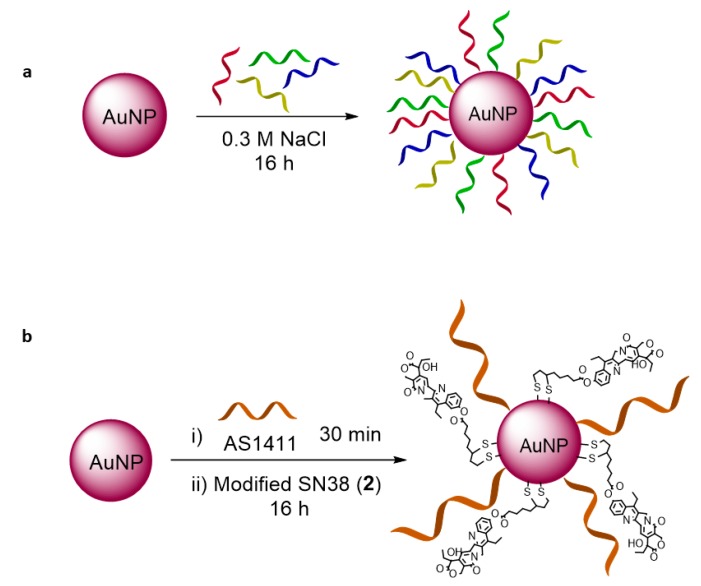
Functionalization of AuNPs with (**a**) DNA mix 2, and (**b**) aptamer AS1411 and modified SN38 (**2**).

Gold nanoparticles were prepared based on the Turkevich method [64] and modified with the oligonucleotides (DNA mix 2 and siRNA mix) and SN38 as described in Section 2.4. Specifically, the bare nanoparticles were incubated with oligonucleotides (DNA mix 2 or siRNA mix) modified with a thiol moiety in the presence of NaCl for 16 h. In the case of the nanoparticle modified with SN38, the particles were previously incubated with the aptamer AS1411 for 30 min, and the modified SN38 (**2**) was then added to the solution (Figure 4). 

**Figure 5:**
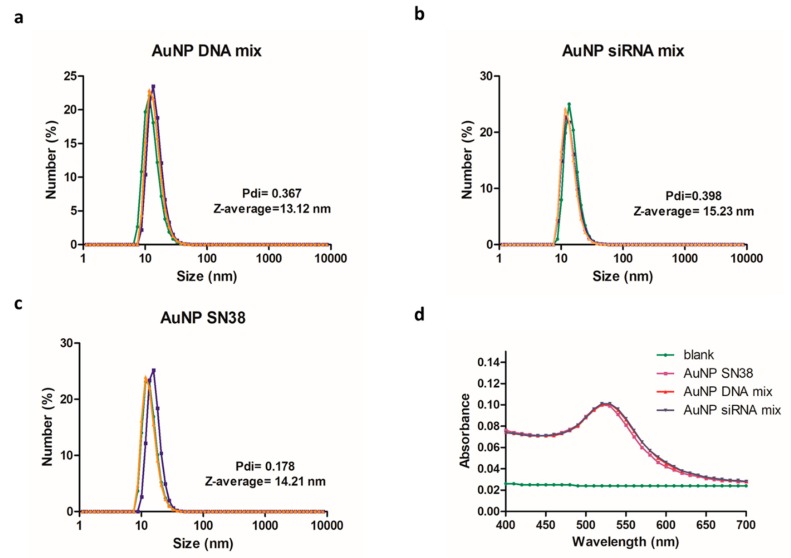
Characterization of AuNPs. (**a**–**c**) Hydrodynamic diameter size (nm) of modified AuNPs and polydispersity index (Pdi). (**d**) Ultraviolet–visible (UV–vis) spectrum where the corresponding plasmon band at 520 nm was noticeable.

Once the unbound material was removed, the sizes of the three formulations (AuNPs DNA mix, AuNPs siRNA mix, and AuNP SN38) were characterized by DLS. The average size of the hydrodynamic diameter was 13–15 nm (Figure 5a–c).

The zeta-potential was carried out to assess the charges in the AuNPs before and after the functionalization using solutions of the samples in water at 8.3 nM. Bare AuNPs had a zeta-potential of −37.9 ± 0.603 mV; AuNP SN38 of −22.1 ± 1.84 mV; AuNP DNA mix of −35 ± 2.72 mV; and AuNP siRNA mix of −35.8 ± 2.35 mV. 

The ultraviolet–visible (UV–vis) spectrum, performed in a plate reader synergy H4 Hybrid reader (BioTEK) at 25 °C, revealed the characteristic plasmon band at 520 nm of AuNPs (Figure 4d).

**Figure 6:**
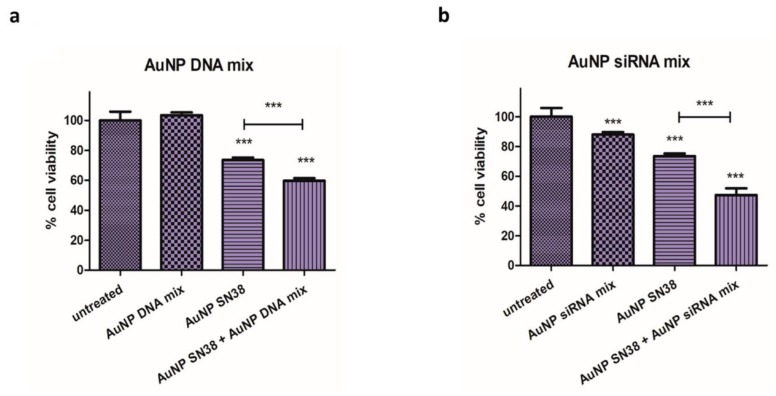
Cell viability assay in Mel 202 cells treated with (**a**) AuNP DNA mix, AuNP SN38, and their combination (AuNP DNA mix + AuNP SN38) (*** *p* < 0.001). (**b**) AuNP siRNA mix, AuNP SN38 and their combination (AuNP siRNA mix + AuNP SN38) (*** *p* < 0.001). Statistical analysis was performed using one-way ANOVA test (each group vs. control).

The activity of functionalized AuNPs was evaluated in Mel 202 cells, where the AuNP DNA mix did not reduce the viability of the cells. When the AuNP SN38 was added the viability was reduced, and this reduction was even higher when used in combination with the AuNP siRNA mix (Figure 6a).

**Figure 7:**
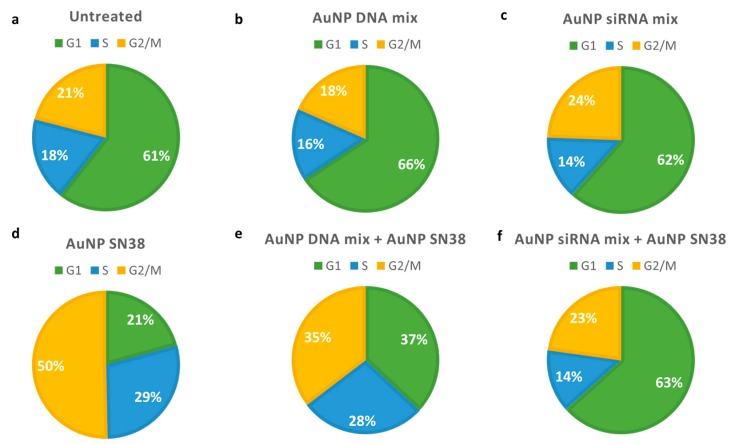
Pie charts describing flow cytometry data in Mel 202 cells. (**a**) Untreated cells; (**b**) Cells treated with 100 μL AuNP DNA mix 2; (**c**) Cells treated with 100 μL AuNPs siRNA mix; (**d**) Cells treated with 25 nM AuNPs SN38; (**e**) Cells treated with 100 μL AuNPs DNA mix 2 and 25 nM AuNP SN38; (**f**) Cells treated with 100 μL AuNPs siRNA mix and 25 nM AuNP SN38. Green: cells in G1 phase; blue: cells in S phase; yellow: cells in G2/M phase.

Cell viability on Mel 202 was reduced after AuNP siRNAs mix treatment, and it was further reduced when AuNP siRNAs were combined with AuNP SN38. (Figure 6b). Interestingly, the cell cycle was affected when SN38 was present, but not when siRNAs were used in the formulation (Figure 7, and Supplementary Figures S5 and S6).

## 4. Discussion

### 4.1. DNA-Based microRNA Mimics

Antisense technology has been used to reduce the expression of mRNAs [64]. It requires the formation of duplexes between the oligonucleotides and the target mRNAs, which usually leads to the degradation of the RNA by ribonuclease H (RNase H). Herein, this reactivity has been exploited using oligonucleotides with the same sequence as four microRNAs downregulated in UM: microRNA-34a, microRNA-182, microRNA-137, and microRNA-144 [24]. These DNA-based microRNA mimics are expected to reduce the tumor behavior of cancer cells [65] or increase their sensitivity to chemotherapeutics [66]. 

To test the effectiveness of the selected DNA-based microRNA mimics, 1, 2, 3 and 4 (Table 1) were transfected individually, in pairs and all together into Mel 202 cells (Figure 1a–c). The effect achieved by single oligonucleotides was negligible, including when combined in pairs. Interestingly, a 30% reduction in viability resulted when all the oligonucleotides were used as a 1:1:1:1 mixture, despite their lower individual concentration compared with the previous two experiments. This reduction might be due to a synergistic activity when combining the inhibitions of the different pathways associated with the microRNAs. 

After this experiment, the effect of the microRNA mimics in the cells was assessed using other techniques. Since the microRNAs selected can reduce the expression of c-Met [27,32,67], the effect of the DNA mix 1 was studied by immunofluorescence. c-Met is a tyrosine kinase receptor implicated in the metastasis and malignancy of UM [28] and is usually overexpressed in UM [68,69]. Remarkably, the images revealed significant changes in fluorescence when the cells were treated with DNA mix 1, which resulted in a 67% reduction in expression (Figure 1e).

### 4.2. Combination Therapy

Since the DNA-based microRNA mimics affect cell viability and c-Met expression, their effect was studied in combination with a chemotherapeutic drug. This approach might prevent the generation of resistance since c-Met is significantly reduced [70]. Here, SN38 was selected as an analog of Irinotecan, which has been recently used in metastatic UM [71].

After assessing the IC50 of the drug in Mel 202 cells (Figure 2a), the effect of the SN38 in combination with DNA mix 1 and the siRNA mix was studied (Figure 2b). A significant reduction in the cell viability (50%) was observed using low concentrations of the drug (25 nM) and DNA mix 1 or the siRNA mix. Remarkably, the effect observed was synergistic according to the method described by Valeriote [72]. In this method, the efficacy of the individual drugs (εA, εB) is compared to the combined treatment (εA + B). It is synergistic if εA + B < (εA × εB)/100; additive, if εA + B = (εA × εB)/100; sub-additive if (εA × εB)/100 < εA + B < εA, provided εA < εB; interfering if εA < εA + B < εB, when εA < εB; or antagonistic, if εB < εA + B, when εA < εB. According to this method, the proposed treatment had a synergistic effect.

This result highlights the potential of this approach, where reprograming the behavior of UM through oligonucleotides allows for a significant reduction of the dose of SN38 to 25 nM; potentially decreasing side effects [73]. 

Furthermore, the reduction of c-Met expression observed in the combination approach could also lead to a reduction of drug resistance, as mentioned previously. 

The effect of this approach was also studied on the cell cycle, which is related to tumor progression and cell death. DNA mix 1 did not modify the cell cycle significantly. In contrast, SN38 promoted a reduction in the G1 phase and an increase in G2 phase (Figure 3c), as previously reported [74,75]. When DNA mix 1 and SN38 were used in combination, the G2 arrest was also increased but less than in the case of SN38 alone (Figure 3c,d), despite the higher decrease in cell viability observed (Figure 2b).

### 4.3. Gold Nanoparticles as Vehicles

As mentioned in the Introduction, the delivery of oligonucleotides and drugs can be problematic for several reasons. The charges of phosphate groups on the oligonucleotides prevent their internalization inside the cells [49,76], and the drug has low solubility in water [17]. Thus, gold nanoparticles were used to overcome these limitations. The conjugation of oligonucleotides was achieved through a thiol modification. Thus, the oligonucleotides were deprotected using TCEP and incubated with the gold nanoparticles, affording stable nanostructures. In the case of SN38, a linker was prepared that allows its easy conjugation to the nanoparticles. However, the first attempts led to the aggregation of the nanostructure. To increase its stability, the aptamer AS1411 was used, which has previously been employed to stabilize nanoparticles [47]. This aptamer can recognize the nucleolin protein present in the membrane of tumoral cells [77] and therefore can be used to increase their internalization. Using this approach, stable AuNPs modified with SN38 were obtained. 

The changes in the zeta-potential confirms that the nanoparticles have been modified. In this regard, the addition of SN38 reduced the negative charge, as expected. In the case of AuNP DNA mix or AuNP siRNA mix we did not observe significant changes in the charge. This is due to the presence of the phosphate groups.

The particles prepared were tested in cell culture, individually and in combination, revealing that when the two types of nanoparticles where used (AuNP DNA mix and AuNP SN38), a higher inhibition was obtained, as observed previously with the oligonucleotides and the free drug.

Furthermore, AuNPs modified with siRNAs instead of DNAs were assessed, since siRNAs inhibit gene expression or mimic microRNAs better than DNA oligonucleotides. In this case, the inhibition by the AuNP siRNA mix was higher than that by the AuNP DNA mix, and it was even higher when combined with SN38. Interestingly, in both cases, the effect was synergistic [72], probably due to their different mechanism of action and the different process and pathways involved in both therapeutics. 

The cell cycle analysis showed a significant effect when AuNP SN38 was used. However, this effect was reduced when combined with AuNP DNA mix, and particularly with AuNP siRNA mix. This result suggests that the DNA or RNA therapy reprogrammed the cells to a state where the G2 arrest of SN38 cannot be detected. In other words, the selected oligonucleotides could lead the cell cycle progression as was previously reported with other microRNAs, such as the microRNA-181 family [78].

## 5. Conclusions

The use of microRNA mimics as therapeutics against uveal melanoma has been evaluated. These DNA-based mimics are not active when used individually or in pairs, but they present significant activity when used in combination. This might be due to the synergy of the sequences selected that interact with different pathways involved in cell survival. Remarkably, this system can be used to increase the sensitivity of Mel 202 cells to SN38, requiring only 25 nM to achieve the same activity of the drug alone at 100 nM. Again, the synergy observed with this combination might be due to the complementary targets of the bioactive molecules (microRNA mimics and SN38). Furthermore, microRNA mimics and SN38 have been conjugated to gold nanoparticles to overcome their inherent limitations for biomedical applications, such as stability, translocation, and solubility. Interestingly, the nanoformulations obtained present an activity comparable to the free bioactive molecules, and the same synergistic effect when combined. Finally, RNA-based mimics have been used in the same way, providing even higher cytotoxic activity. The unique combination of oligonucleotides employed here might be reprogramming the cancer cells, making them more susceptible to SN38.

## Figures and Tables

**Table 1:** Oligonucleotides (DNA and RNA) used in this study.

Entry	Oligonucleotides	Sequence
1	DNA-34a	5′-TGGCAGTGTCTTAGCTGGTTGTTAAAAA-3′
2	DNA-182	5′-TTTGGCAATGGTAGAACTCACACTAAAAA-3′
3	DNA-137	5′-TTATTGCTTAAGAATACGCGTAGAAAAA-3′
4	DNA-144	5′-GGATATCATCATATACTGTAAGAAAAA-3′
5	DNA-34a	5′-TGGCAGTGTCTTAGCTGGTTGTTAAAAA–Thiol-3′
6	DNA-182	5′-TTTGGCAATGGTAGAACTCACACTAAAAA–Thiol-3′
7	DNA-137	5′-TTATTGCTTAAGAATACGCGTAGAAAAA–Thiol-3′
8	DNA-144	5′-GGATATCATCATATACTGTAAGAAAAA–Thiol-3′
9	RNA-34a pass	5′-Thiol–AAAAAArCrArArCrCrArGrCrUrArArGrArCrArCrUrGrCrCrU-3′
10	RNA-34a guide	5′-rUrGrGrCrArGrUrGrUrCrUrUrArGrCrUrGrGrUrUrGrU-3′
11	RNA-182 pass	5′-Thiol–AAAAAArGrUrGrUrGrArGrUrUrCrUrArCrCrArUrUrGrCrCrArArA-3′
12	RNA-182 guide	5′-rUrUrUrGrGrCrArArUrGrGrUrArGrArArCrUrCrArCrArCrU-3′
13	RNA-137 pass	5′-Thiol–AAAAACUACGCGUAUUCUUAAGCAAUAA-3′
14	RNA-137 guide	5′-rUrUrArUrUrGrCrUrUrArArGrArArUrArCrGrCrGrUrArG-3′
15	RNA-144 pass	5′-Thiol–AAAAArCrUrUrArCrArGrUrArUrArUrGrArUrGrArUrArUrCrC-3′
16	RNA-144 guide	5′-rGrGrArUrArUrCrArUrCrArUrArUrArCrUrGrUrArArG-3′
17	AS1411 [47]	5′-GGTGGTGGTGGTTGTGGTGGTGGTGGTTTTTT–Dithiolane-3′

**Table 2:** Modification of gold nanoparticles (AuNPs).

Nanoparticle	Reagents Used in the Preparation
**AuNP DNA mix**	500 μL DNA mix 2
**AuNP siRNA mix**	500 μL siRNA mix
**AuNP SN38**	4.06 μL AS1411 (492 μM) + 5 μL SN38 (2 mM in DMSO)

## Supplementary Materials

The following are available online at http://www.mdpi.com/2313-7673/3/4/28/s1. Figure S1: TEM analysis of AuNPs, Figure S2: ^1^H NMR of compound **1**, Figure S3: ^1^H NMR of compound **2**, Figure S4: ^13^C NMR of compound **2**, Figure S5: Cell cycle analysis, Figure S6: Distribution of Mel 202 cells treated with AuNPs by flow cytometry.
