# Supplementary material for: Reprogramming Cells for Synergistic Combination Therapy with Nanotherapeutics against Uveal Melanoma
Source: Biomimetics (Basel). 2018 Sep 25;3(4):28. doi: 10.3390/biomimetics3040028 (PMC6352695; doi:10.3390/biomimetics3040028)
Supplement: Supplementary file 1 [file biomimetics-03-00028-s001.zip › biomimetics-332633-supplementary.pdf]

Biomimetics **2018**, *3*, 28

Supplementary Materials: Reprogramming Cells for Synergistic Combination Therapy with Nanotherapeutics against Uveal Melanoma

Paula Milán Rois, Alfonso Latorre, Ciro Rodriguez Diaz, Álvaro del Moral and Álvaro Somoza*

Instituto Madrileño de Estudios Avanzados en Nanociencia (IMDEA Nanociencia), Centro Nacional de Biotecnología (CNB-CSIC) Associated Unit, Faraday 9, 28049 Madrid, Spain; paula.milan@imdea.org (P.M.R.); alfonso.latorre@imdea.org (A.L.); ciro.rodriguez@imdea.org (C.R.D.); delmoilex@gmail.com (Á.d.M.)

* Correspondence: alvaro.somoza@imdea.org; Tel.: +34-912-998-856

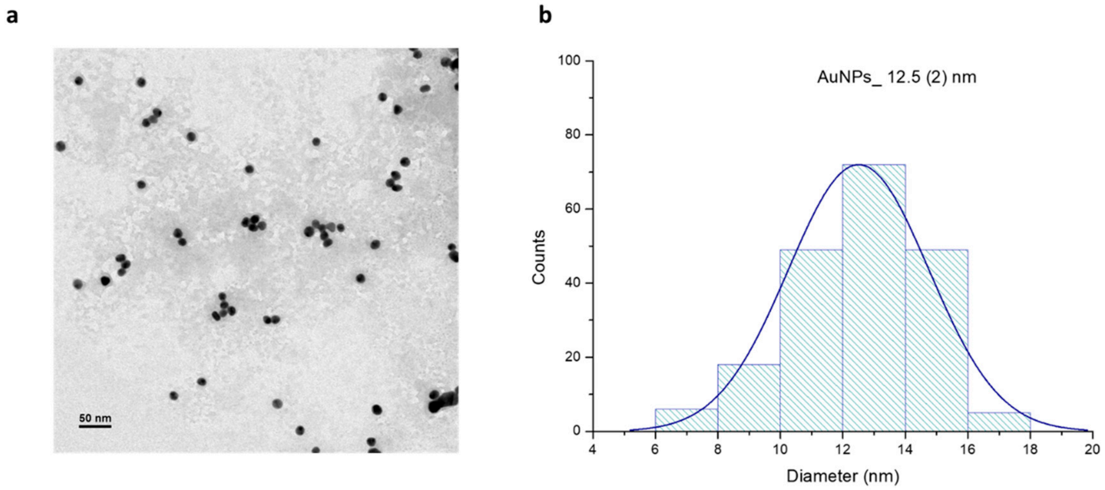

Figure S1. TEM analysis of AuNPs. (**a**) TEM images of AuNPs. Homogeneous size was observed. (**b**) Histogram of the sizes of the AuNP obtained. The diameter of AuNPs was 12.5 ± 2 nm.

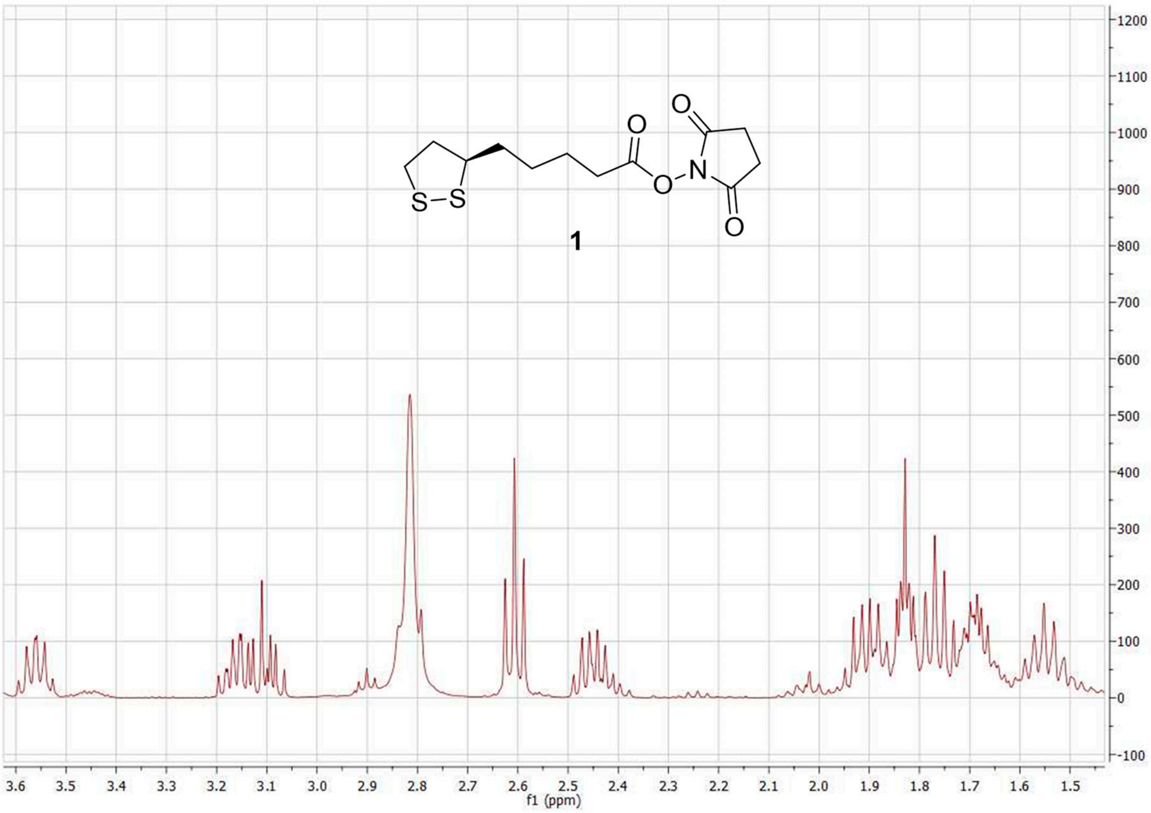

Figure S2. ^{1}H NMR of compound **1.**

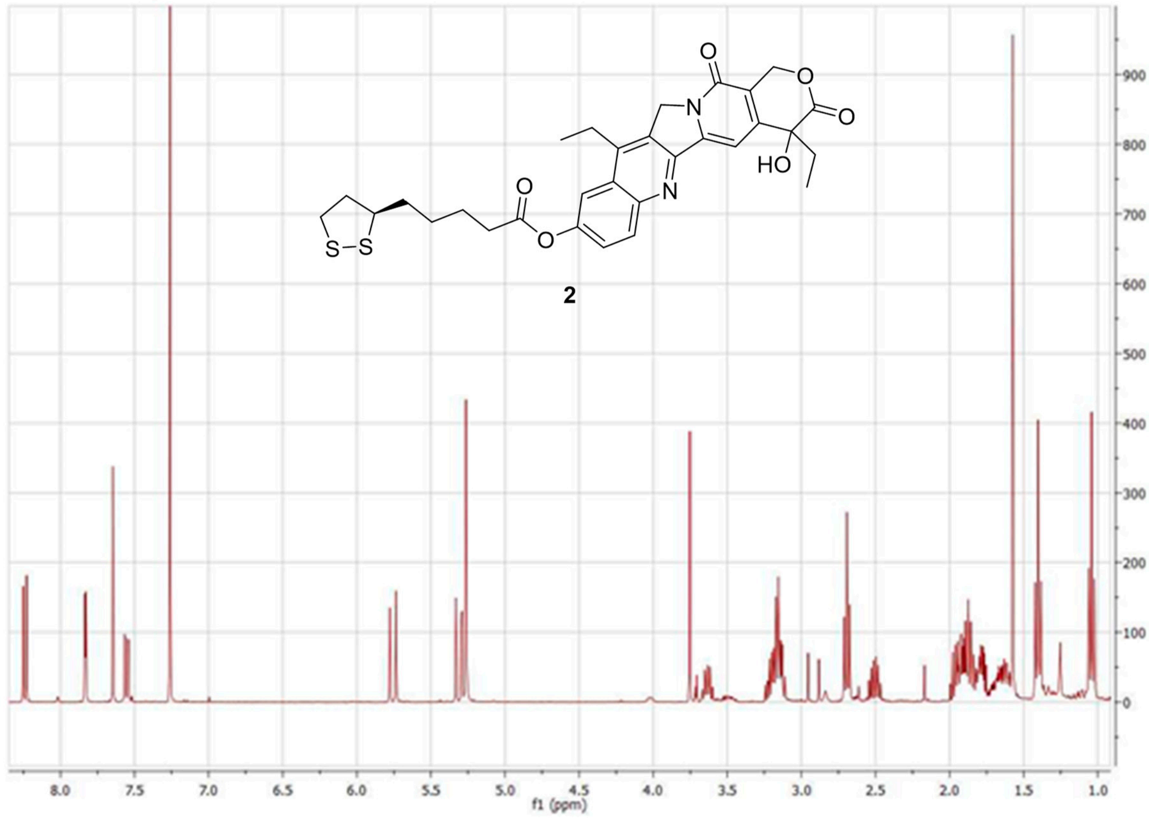

Figure S3. ^{1}H NMR of compound **2**.

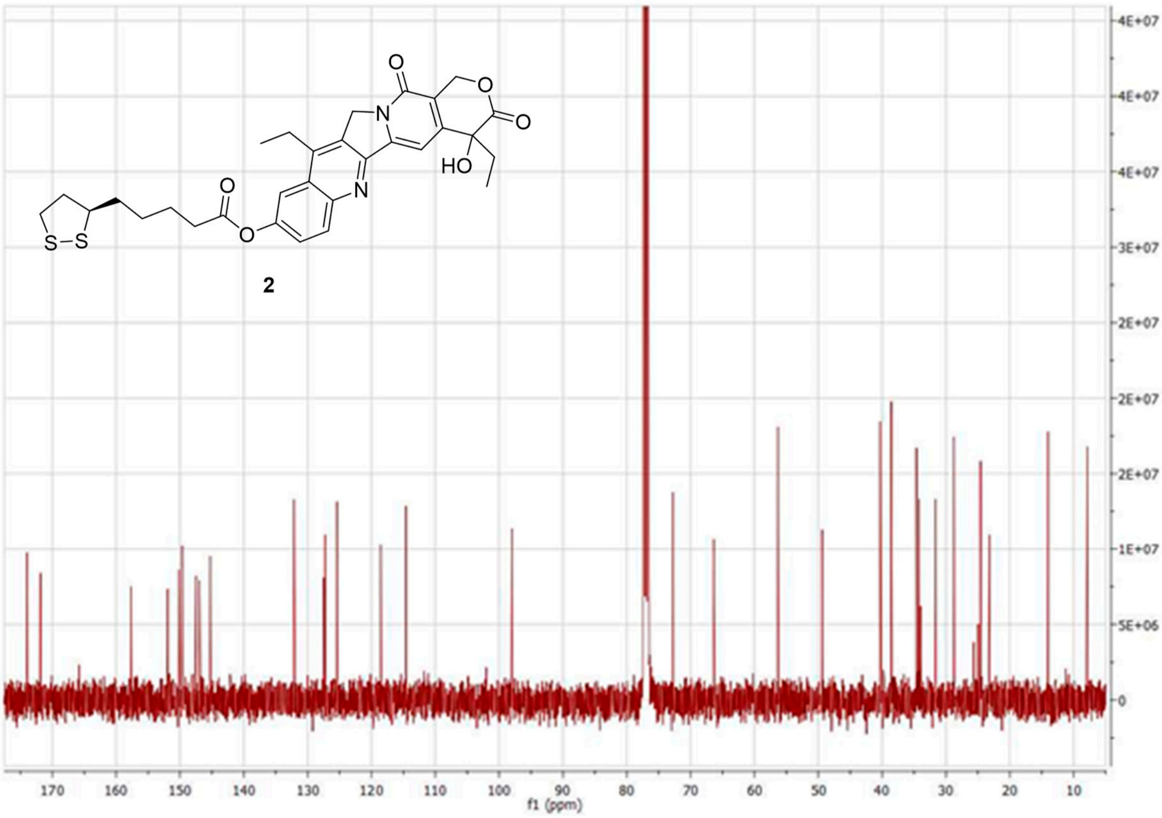

Figure S4. ^{13}C NMR of compound **2**.

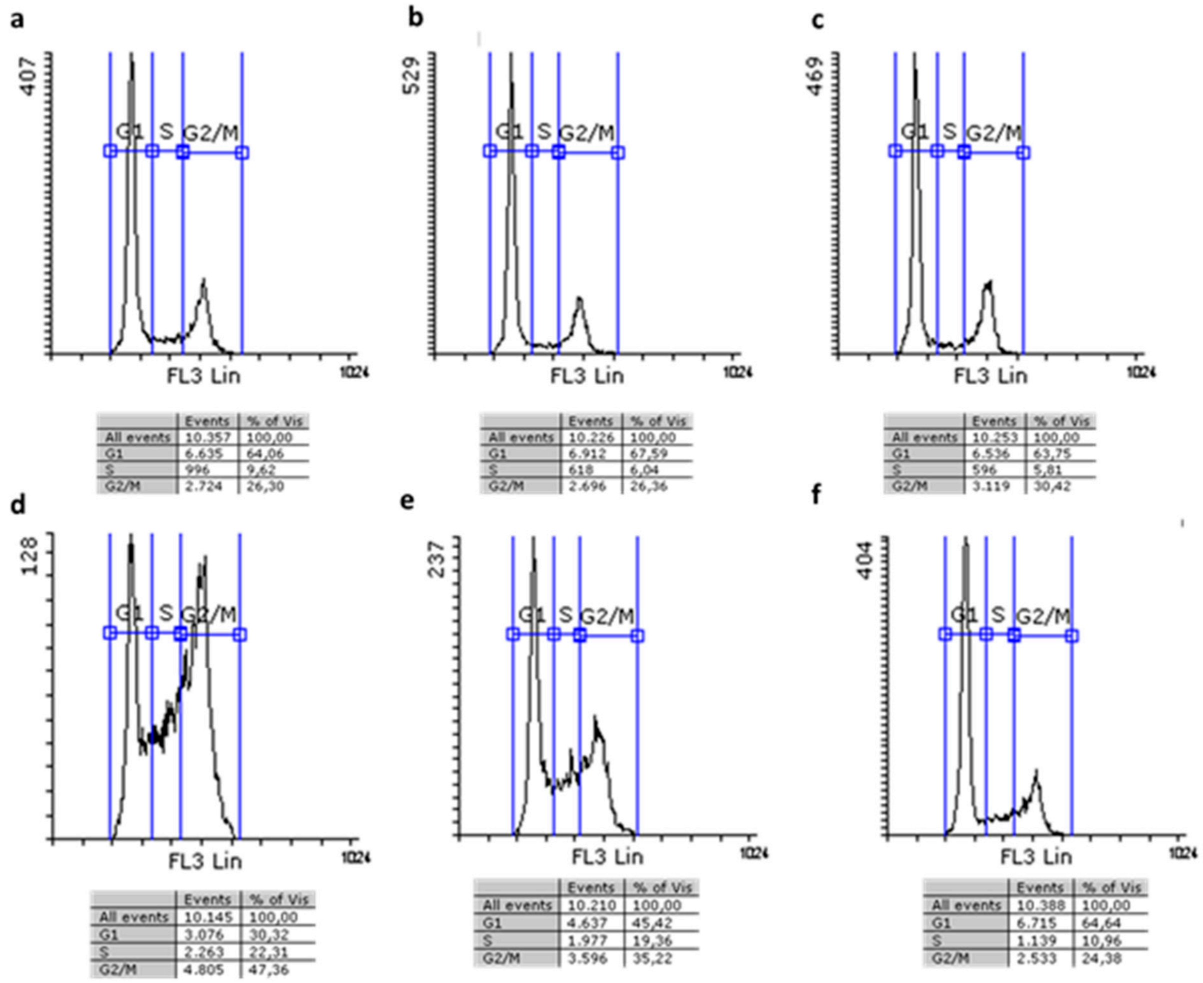

Figure S5. Cell cycle analysis. (**a**) Untreated condition; (**b**) AuNP DNA mix; (**c**) AuNP RNA mix; (**d**) AuNP SN38; (**e**) AuNP DNA mix + AuNP SN38; (f) AuNP RNA mix + AuNP SN38.

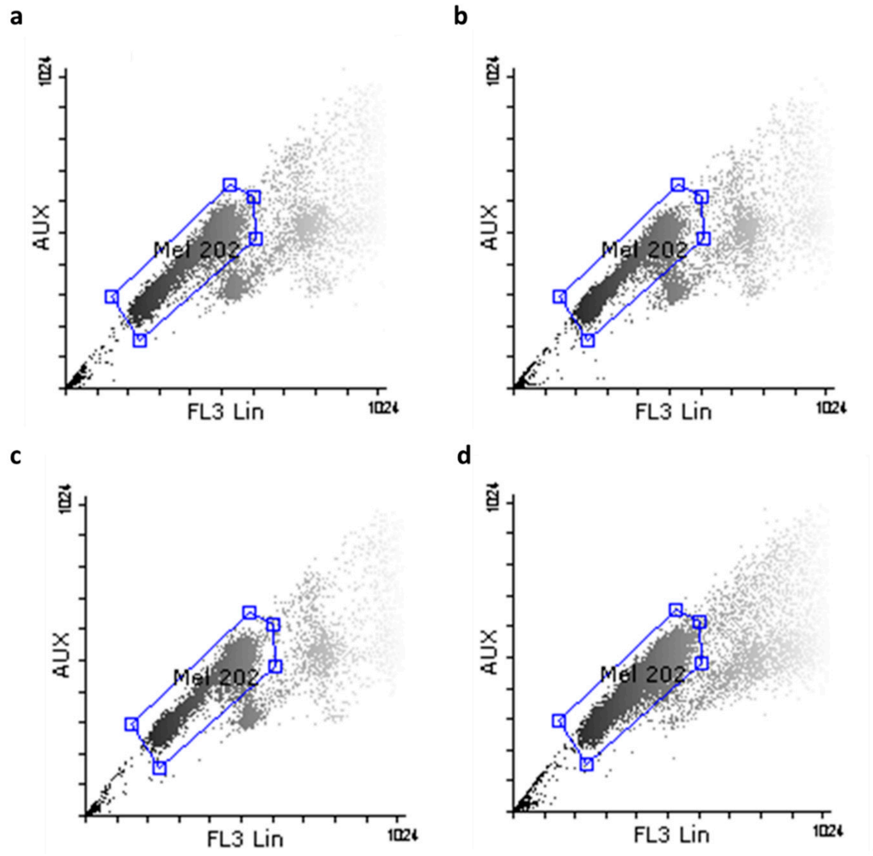

Figure S6. Distribution of Mel 202 cells treated with AuNPs by flow cytometry. The gate indicates the complete single cells selected for the analysis (neither membrane residues nor doublet cells). (**a**) AuNPs; (**b**) AuNPs DNA mix; (**c**) AuNPs siRNA mix; (**d**) AuNPs SN38.
